# CD44^+^ fibroblasts increases breast cancer cell survival and drug resistance *via* IGF2BP3‐CD44‐IGF2 signalling

**DOI:** 10.1111/jcmm.13118

**Published:** 2017-05-18

**Authors:** Yonglei Liu, Conghui Yu, Yonggang Wu, Xiangjun Sun, Quanping Su, Cuiping You, Hongwu Xin

**Affiliations:** ^1^ Research Center Linyi People's Hospital Shandong China; ^2^ Zhongshan Hospital Fudan University Shanghai China; ^3^ The First Peoples' Hospital School of Clinical Medicine Yangtze University Jingzhou Hubei China; ^4^ Laboratory of Oncology Center for Molecular Medicine School of Medicine Yangtze University Jingzhou Hubei China; ^5^ Department of Hepatobilinary Surgery The General Hospital of Beijing Military Region of China Beijing China; ^6^ Department of Orthopedics Bayannaoer City Hospital Bayannaoer City Inner Mongolia China; ^7^ Department of Surgery Linyi People's Hospital Shandong China

**Keywords:** breast cancer, IGF2BP3, IGF2, fibroblasts

## Abstract

CD44, a cell adhesion protein, involves in various process in cancer such as cell survival and metastasis. Most researches on CD44 in cancer focus on cancer cells. Recently, it is found that CD44 expression is high in fibroblasts of tumour microenvironment. However, its role in communication between fibroblasts and breast cancer cells is seldom known. In this study, CD44‐positive (CD44^+^Fbs) and CD44‐negative carcinoma‐associated fibroblasts (CD44^−^Fbs) were isolated and cocultured with breast cancer cells for analysis of cell survival and drug resistance. We found that CD44^+^Fbs promoted breast cancer cell survival and paclitaxel resistance and inhibited paclitaxel‐induced apoptosis. Our further research for the molecular mechanism showed that IGF2BP3 bound to CD44 mRNA and enhanced CD44 expression, which increased IGF2 levels of fibroblasts and then stimulated breast cancer cell proliferation and drug resistance. IGF2 was found to activate Hedgehog signal pathway in breast cancer cells. In conclusion, the results illustrated that in CD44^+^Fbs, binding of IGF2BP3 and CD44 promotes IGF2 expression and then accelerates breast cancer cell proliferation, survival and induced chemotherapy resistance likely by activating Hedgehog signal pathways.

## Introduction

The tumour microenvironment plays very important roles in the development and progression of cancer. Tumour tissues consist of various cells and non‐cell components. The cell components include cancer cells, fibroblasts, endothelial cells, pericytes, immune cells and various bone marrow‐derived progenitor cells 1, 2. Fibroblasts are the most abundant cells in tumour stroma. The activated cancer‐associated fibroblasts in the cancer niche build a permissive and supportive microenvironment for tumour development and play important roles in cancer progression including metabolism, metastasis, proliferation, anti‐apoptosis, angiogenesis and chemoresistance by interaction with cancer cells or other cells 3, 4, 5. Fibroblasts could affect behaviours of cancer cells by activating signal pathways, miRNAs and soluble molecules and so on.

Growth factors are critical signalling mediators, which involves the communication between the cancer cells and fibroblasts through binding to their receptors 6, 7. Cell survival, proliferation, obtaining stem cell properties, ECM attachment, adhesion, metastasis in tissues or other organs including proteolysis of the basement membrane, locomotion and colony formation can be changed according to the signals 8, 9, 10, 11. Fibroblasts secrete a variety of growth factors that modulate cancer progression and metastasis 1, 2, 3, 4, 5. Reports showed that fibroblasts involve in the crosstalk of cancer cells and fibroblasts by secreting TGF‐β, FGF, PDGF, IGF and others 4, 5. It is reported that IGF2 was up‐regulated in breast cancer stroma 12, 13, 14; however, its role and molecular mechanism in the crosstalk between fibroblasts and cancer cells are still unknown.

Recently, in the period of studying roles of fibroblasts on cancer cells, we found that CD44 expression level was very high in fibroblasts; thus, we suggested that the fibroblasts with high CD44 expression may play important roles in the effect on breast cancer cells. Therefore, CD44^+^Fbs and CD44^−^Fbs were sorted out to investigate their roles and molecular mechanisms in communication between fibroblasts and breast cancer cells.

## Materials and methods

### Cell lines and culture

The breast cancer cell lines including MCF‐7, MDA‐231, SKBR3, MDA‐435, ZR75B and BT474 were originally purchased from American Type Culture Collection (ATCC, Manassas, VA, USA) and maintained in Dulbecco's modified Eagle's medium containing 10% foetal bovine serum, 100 units/ml penicillin and 100 μg/ml streptomycin. Hs578Bst fibroblasts were obtained from ATCC and maintained in Hybri‐Care Medium (ATCC) with 30 ng/ml EGF, 100 units/ml penicillin and 100 μg/ml streptomycin. Fibroblasts (Fbs) were generated in the conditioned medium from cocultured Hs578Bst and breast cancer cells for 3‐5 days. The cell lines were cultured in a humidified atmosphere of 95% air and 5% CO_2_ at 37°C.

### Coculturing of breast cancer cells and fibroblasts, and conditioned medium preparation

Fibroblasts were cocultured with breast cancer cells with the ratio at 1:1 using Transwell coculture system. Cells were cultured in DMEM/F12 media with 10% FBS supplemented with 10% FBS in a 37°C humidified incubator with an atmosphere of 5% CO_2_ and 95% air for 24 hrs, and then washed for three times with PBS and finally cultured in 3 ml serum‐free DMEM/F12 media for 2 hrs. Conditioned medium was collected and filtered through a 0.22‐μm filter (Merck Millipore, Billerica, Massachusetts, USA) to remove cellular debris.

### Reagents

Antibodies against CD44 and IGF2BP3 were purchased from Santa Cruz (Dallas, TX, USA). Antibodies of Bax, bcl‐2, caspase‐3, Gli2, Hhip, Ptch1 and Ptch were obtained from Cell Signaling Technology. All other chemicals were purchased from Sigma‐Aldrich (Saint Louis, MO, USA). Recombinant human IGF2 was purchased from R&D (Minneapolis, MN, USA).

### Real‐time RT‐PCR

Total RNA was extracted from the cells with the indicated treatment using TRIzol reagent (Invitrogen, Carlsbad, CA, USA) according to the manufacturer's protocol. RNA was quantified and performed for real‐time RT‐PCR analysis. The relative mRNA levels were calculated by comparing Ct values of the samples with those of the reference; all data were normalized to the internal control GAPDH.

### IGF2BP3 overexpression

IGF2BP3 (NM_006547.2) from Hs578Bst fibroblasts was amplified using PCR and then cloned into lentivirus vector pKLR. The lentivirus carrying IGF2BP3 was produced in 293T cells transfected with IGF2BP3 lentivirus vector and the packing plasmids. The lentivirus was transfected into the fibroblasts for experimental analysis.

### Dual luciferase assay

CD44 promoter was amplified and cloned in PGL3 (Promega, Madison, WI, USA) vector and transfected into breast cancer cells in Opti‐MEM medium using Lipofectamine‐2000 according to the manufacturer's protocol (Invitrogen). The medium was changed after transfection for 5 hrs, and the cells were incubated at 37 °C for the indicated time. Luciferase activity was detected using Dual Luciferase Assay System (Promega) with a Sirius luminometer (Berthold Detection System).

### ELISA for the measurement of IGF‐2 in conditioned medium

Conditioned medium was employed to quantitatively measure IGF2 using a sandwich enzymatic method with specific anti‐IGF2 antibodies (R&D Systems). The supernatant was collected and used for ELISA according to the manufacturer's guidelines. Briefly, cells were grown to confluence in media supplemented with 10% FBS which was then replaced with serum‐free medium. A total of 200 μl of cell supernatant was incubated with 50 μl of assay diluent for 2 hrs at room temperature in a 96‐well plate coated with a monoclonal antibody against IGF2. After three washes, a conjugate consisting of IGF2 antibody and horseradish peroxidase was incubated for 2 hrs at room temperature. After addition of a colour reagent, absorbance was measured at 450 nm in a Thermo‐Max microplate reader. For standardization, serial dilutions of recombinant human IGF2 were assayed at the same time.

### IGF‐2 blocking peptide

IGF2 antibody (Center R54) blocking peptide was from Abgent Biotech (SuZhou, China). This peptide was used to block IGF2 binding to its target.

### Western blot analysis

Cells were lysed in a lysis buffer containing 50 mmol/l Tris‐HCl, pH 7.4, 150 mmol/l NaCl, 0.5% NP40, 50 mmol/l NaF, 1 mmol/l Na_3_VO_4_, 1 mmol/l phenylmethylsulfonyl fluoride, 25 μg/ml leupeptin and 25 μg/ml aprotinin and clarified by centrifugation (14,000 × *g* for 30 min. at 4°C). The protein concentration was determined using the Bradford Coomassie blue method (Pierce Chemical Corp., Dallas, TX, USA). Whole‐cell lysates were separated by sodium dodecyl sulphate (SDS)‐PAGE, transferred onto nitrocellulose and probed with various primary antibodies and horseradish peroxidase‐labelled secondary antibodies. The signals were visualized with an enhanced chemiluminescence detection kit (Promega).

### ShRNA lentivirus vector construction

ShRNA lentiviral particle delivery system was used to generate IGF2BP3 shRNA and IGF2BP3‐silenced tumour cell lines according to the manufacturer's instructions (Sigma‐Aldrich). The lentiviral particles were purchased from Sigma‐Aldrich. After selection under puromycin (1 μg/ml), the knocking down effect in the drug‐resistant cells was evaluated by Western blot.

### Cell proliferation assay

Cells were cultured in 24‐well plates with low‐glucose (1 g/l), low‐serum (0.5% FBS) medium (0.5 ml/well) at 37°C. Following the indicated treatments, 10 mg/ml methylthiazolyldiphenyl‐tetrazolium bromide (MTT) was added (50 μl/well), and the cells were incubated for an additional 2 hrs. The cells were then lysed with a lysis buffer (500 μl/well) containing 20% sodium dodecyl sulphate (SDS) in dimethyl formamide/H_2_O (1:1, v/v; pH 4.7) at 37°C for at least 6 hrs. The relative number of surviving cells in each group was determined by measuring the optical density (OD) of the cell lysates at an absorbance wavelength of 570 nm.

### Cell colony formation

The cells were harvested, sparsely plated and cultured under the normal condition. The medium underwent the replacement at three‐day intervals. And then the cells were fixed in 90% ethanol and stained with crystal violet and colonies consisting of at least 50 cells were counted after about 2 weeks.

### Cell cycle

In 2 ml culture medium, 2 × 10^5^ cells/well (6‐well plate) were seeded, and cultured for the indicated time before collection. The cells were stabilized with 75% ethanol for 24 hrs, dyed with PI and analysed with ModFit of flow cytometry.

### Cell apoptosis

For apoptosis assay, the Annexin V straining was quantified by flow cytometry. The cells were plated on a 6‐well plate, transfected with the indicated plasmids or siRNA or treated with IGF2 at 24 hrs later, and the complete growth medium was changed to growth medium without serum. At another 24 hrs later, the cells were collected, washed in cold PBS twice and resuspended in 1× binding buffer at a concentration of 1 × 10^6^ cells/ml. After that, the cells in 100 μl solution were transferred to a 5‐ml culture tube, with 5 μl Annexin V‐FITC and 5 μl PI (BD Biosciences, San Jose, CA, USA) added, and gently vortexed and incubated for 15 min. at RT in the dark. And finally, 400 μl 1× binding buffer was added to each tube to be analysed by flow cytometry within one hour.

### Statistics

Data were analysed by SPSS 13.0 software and presented as mean ± S.E. of at least three independent experiments. Two‐tailed Student's *t*‐test was used for comparisons of two independent groups. *P *<* *0.05 was considered statistically significant.

## Results

### CD44^+^Fbs promotes breast cancer cell survival

**Figure 1:**
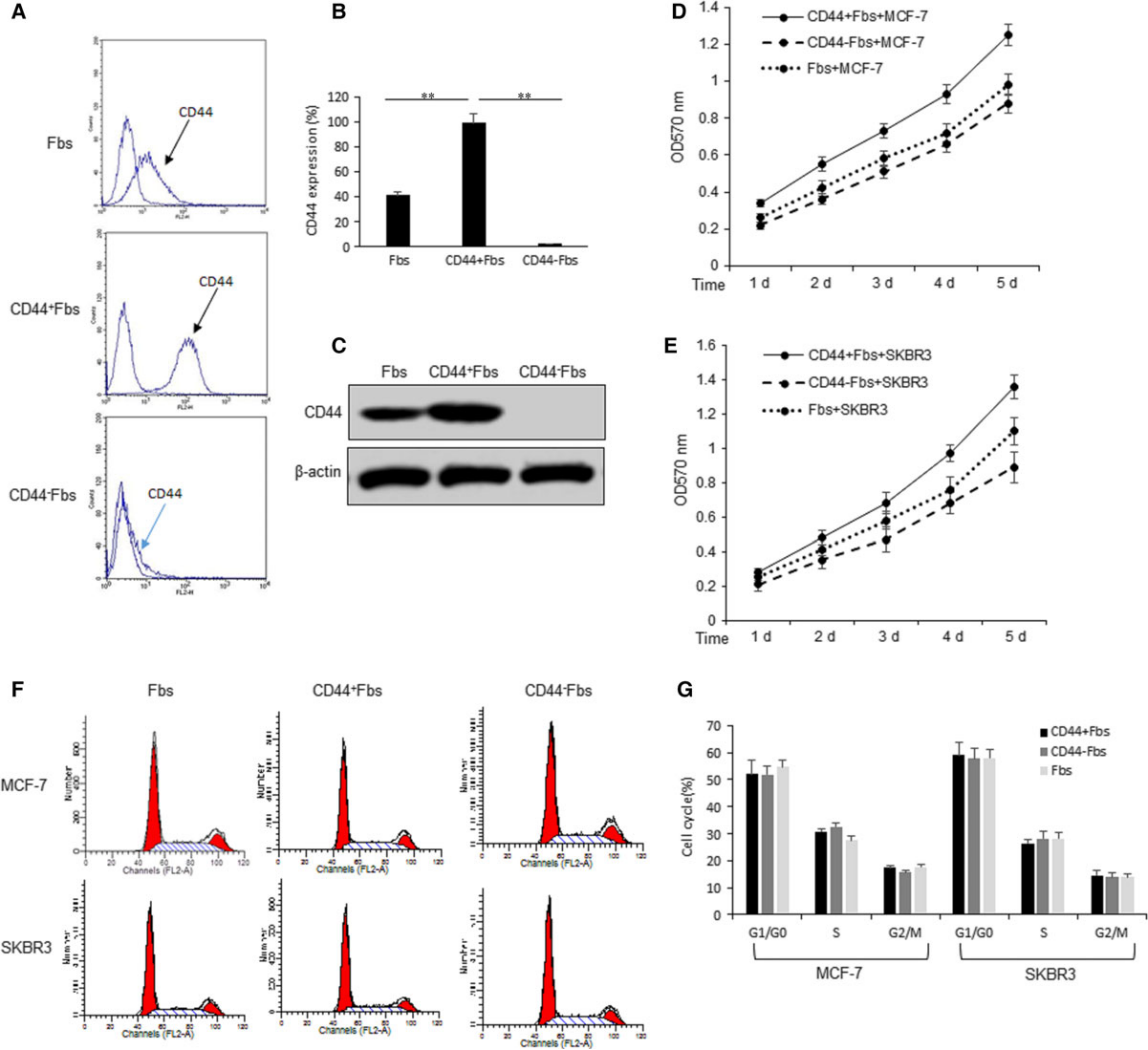
CD44^+^CAFs promote breast cancer cell proliferation. (**A**) CD44 expression in the isolated CD44^+^CAFs or CD44^−^CAFs by flow cytometry. Hs578Bst fibroblasts were treated with the conditioned medium cocultured with MCF‐7 cells and Hs578Bst fibroblasts for 3 days and then sorted out by flow cytometry. (**B**) Data analysis from A. (**C**) CD44 expression in the isolated CD44^+^CAFs or CD44^−^CAFs by Western blotting. (**D**–**E**) Cell proliferation analysis in MCF‐7 and SKBR3 cells treated with CD44^+^CAFs or CD44^−^CAFs by MTT assay. MCF‐7 and SKBR3 cells or CD44^+^ CAFs or CD44^−^CAFs were cocultured using Transwell system. Fibroblasts were in the up chamber and breast cancer cells were in the down chamber. Breast cancer cells were collected to seed and cultured in 96‐well plates in the presence of the medium from the cocultured fibroblasts and cancer cells for MTT assay. (**F**) Cell cycle analysis by flow cytometry in MCF‐7 and SKBR3 cells treated with CD44^+^CAFs and CD44^−^CAFs. Breast cancer cells were treated with the conditioned medium from the cocultured CD44^+^CAFs or CD44^−^CAFs with MCF‐7 or SKBR3 cells. (**G**) Data analysis from F. The data presented are shown as means ± S.D. collected from three independent experiments. **P <* *0.01.

To investigate the role of CD44^+^Fbs on breast cancer cells, CD44^+^Fbs and CD44^−^Fbs were sorted out by flow cytometry. As shown in Figure 1A and B, CD44 expression was very high in CD44^+^Fbs. The result was confirmed using Western blotting analysis (Fig. 1C). Next, we used the Transwell coculture system to culture CD44^+^Fbs and CD44^−^Fbs with MCF‐7 cells and then MCF‐7 cells were seeded in 96‐well plates for MTT assay. It was found that MCF‐7 cells in the presence of conditioned medium of cocultured CD44^+^Fbs and MCF‐7 cells showed higher cell survival ability (Fig. 1D). SKBR3 was also used to verify the data, and the result indicated that cell survival ability became of much higher with coculturing CD44^+^Fbs and SKBR3 cells than CD44^−^Fbs and SKBR3 cells (Fig. 1E). Cell cycle distribution was studied in MCF‐7 and SKBR3 cells with coculturing with CD44^+^Fbs or CD44^−^Fbs. We found that there had no significant changes in the G1/G0, S and G2/M phases (Fig. 1F and G). These results support that the increased cell numbers with CD44^+^Fbs are likely a result of increased cell survival.

### CD44^+^Fbs increase breast cancer cell drug resistance *via* increasing survival

**Figure 2:**
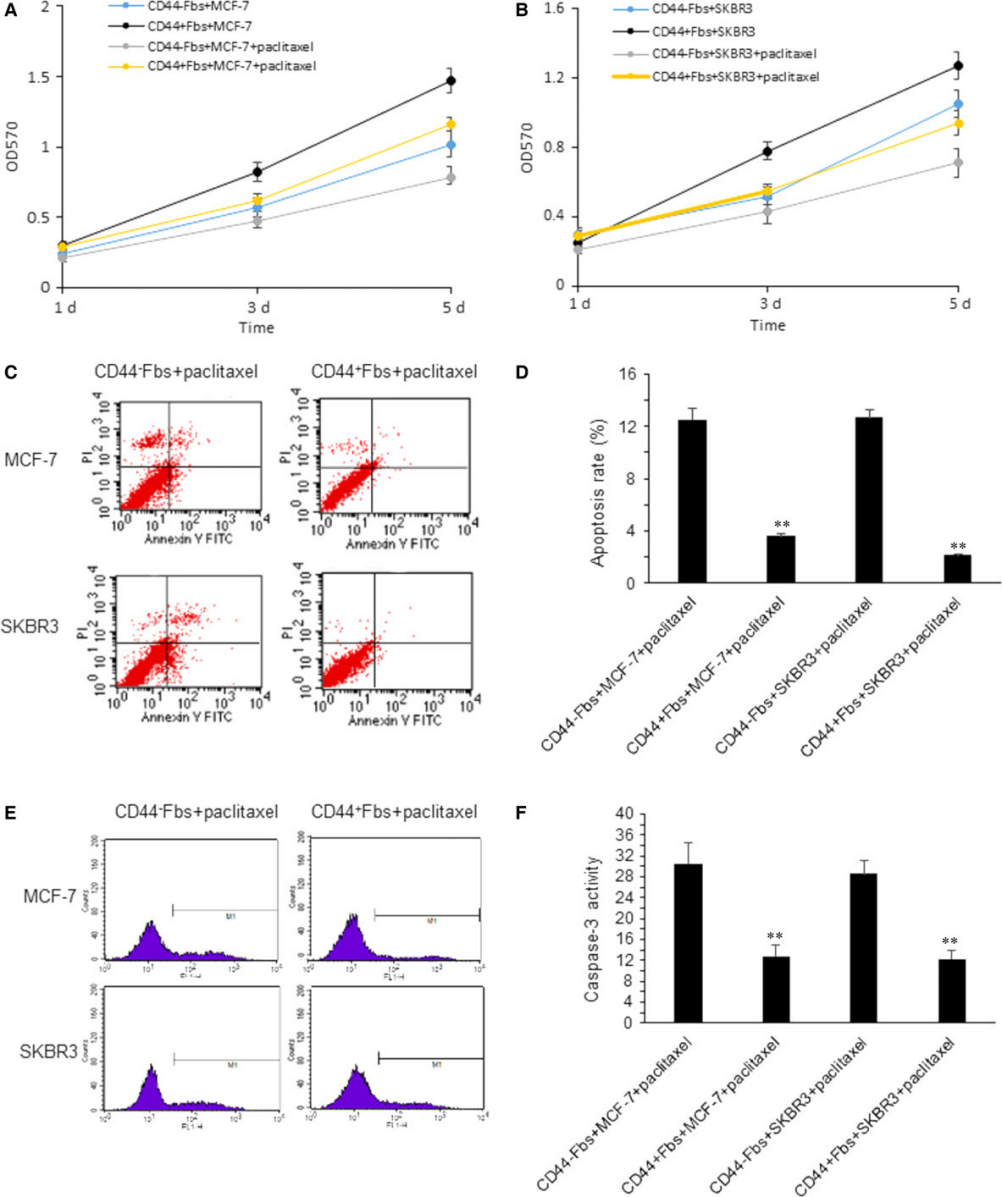
CD44^+^CAFs make breast cancer cell resistant to the drugs. (**A**–**B**) MCF‐7 and SKBR3 cells were cocultured with CD44^+^CAFs and CD44^−^CAFs in Transwell coculture system and exposed to paclitaxel. Cell growth was measured by MTT assay. (**C**–**D**) MCF‐7 and SKBR3 cells were cocultured with CD44^+^CAFs and CD44^−^CAFs in Transwell coculture system and exposed to paclitaxel. Cells were labelled with Annexin V and apoptosis was assayed by flow cytometry. (**E**–**F**) MCF‐7 and SKBR3 cells were cocultured with CD44^+^CAFs and CD44^−^CAFs in Transwell coculture system and exposed to paclitaxel. Cells were labelled with caspase‐3 and apoptosis was assayed by flow cytometry. The data presented are shown as means ± S.D. collected from three independent experiments. *P <* *0.05, **P <* *0.01.

Fibroblasts could induce drug resistance of cancer cells 2, 3, 4. Here, to know whether there is difference in CD44^+^Fbs and CD44^−^Fbs on drug resistance in breast cancer cells, MCF‐7 and SKBR3 cells were exposed to paclitaxel, and then examined the cell survival rate of days 1, 3 and 5. The results indicated that CD44^+^Fbs could make breast cancer cells more proliferating than CD44^−^Fbs (Fig. 2A and B). Cell apoptosis was also examined in the MCF‐7 cells with coculturing CD44^+^Fbs and CD44^−^Fbs and then with paclitaxel treatment for 24 hrs. It was shown that MCF‐7 cells with coculturing CD44^−^Fbs showed more apoptosis rate, so did SKBR3 cells (Fig. 2C and D). Caspase activity was inhibited in MCF‐7 and SKBR3 cells with coculturing CD44^+^Fbs (Fig. 2E and F).

### IGF2BP3 *via* CD44 promotes CD44^+^Fbs to produce more IGF2

**Figure 3:**
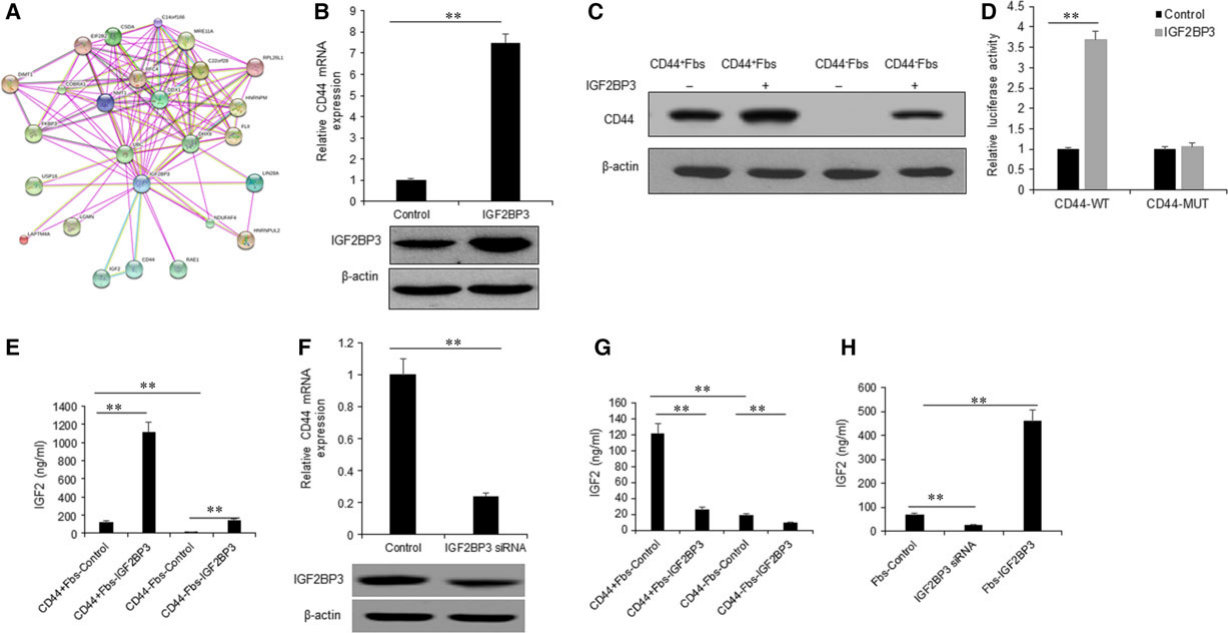
IGF2BP3 promotes CD44^+^CAFs to produce more IGF2. (**A**) The map shows that CD44 is related to IGF2BP3 and IGF2. (**B**) CD44 mRNA expression was enhanced in the fibroblasts with IGF2BP3 overexpression. Lower panel shows the IGF2BP3 expression in the Hs578Bst fibroblasts with IGF2BP3 overexpression. (**C**) CD44 protein levels were enhanced in the fibroblasts with IGF2BP3 overexpression using Western blotting. (**D**) Luciferase assay was used to examine CD44 3′UTR promoter activity. (**E**) CD44^+^CAFs and CD44^−^CAFs were transfected with IGF2BP3 to examine IGF2 by ELISA. (**F**) CD44 mRNA expression decreased in the Hs578Bst fibroblasts with IGF2BP3 knocking down. Lower panel shows the IGF2BP3 expression in the fibroblasts with IGF2BP3 siRNAs. (**G**) CD44^+^CAFs and CD44^−^CAFs were transfected with IGF2BP3 siRNA to examine IGF2 by ELISA. (**H**) Other fibroblasts were transfected with IGF2BP3 to examine IGF2 by ELISA. The data presented are shown as means ± S.D. collected from three independent experiments. **P <* *0.01.

IGF2BP3 (insulin‐like growth factor 2 mRNA‐binding protein 3) was a potential protein that regulates CD44 expression by binding CD44 mRNA (Fig. 3A). When Hs578Bst fibroblasts were transfected with IGF2BP3, CD44 mRNA and protein were up‐regulated (Fig. 3B and C). Next, luciferase assay was used to verify that IGF2BP3 regulates CD44 promoter activity and the result showed that overexpression of IGF2BP3 increased wild‐type CD44 promoter (CD44‐WT) activity, but not the mutated CD44 promoter (CD44‐MUT) in Hs578Bst fibroblasts (Fig. 3D). IGF2BP3 is related to IGF2 induction, CD44^+^Fbs and CD44^−^Fbs were cotransfected with IGF2BP3 and then the conditioned medium was collected for IGF2 measurement. The result indicated that IGF2 levels were increased in the conditioned medium of CD44^+^Fbs with IGF2BP3 overexpression (Fig. 3E). When IGF2BP3 was knocked down, CD44 mRNA decreased in Hs578Bst fibroblasts (Fig. 3F). In CD44^+^Fbs and CD44^−^Fbs with IGF2BP3 knocking down, IGF2 decreased (Fig. 3G). To confirm the results, another CAF cell lines were transfected with IGF2BP3 and then IGF2 in the conditioned medium was measured. It was verified that IGF2 increased in fibroblasts (Fig. 3H).

### Role of IGF2 on cell proliferation and drug resistance in the breast cancer cells

**Figure 4:**
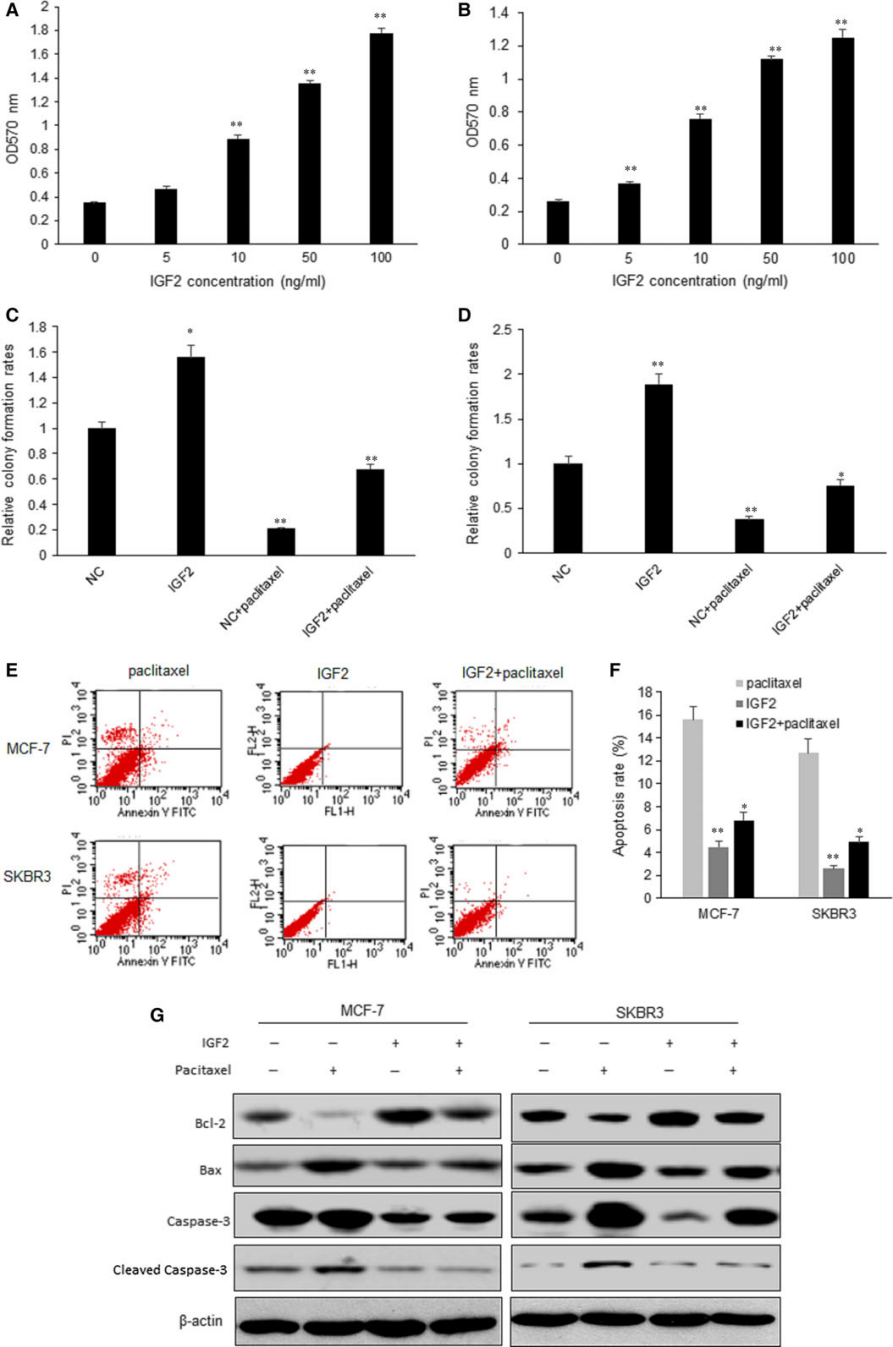
Role of IGF2 on cell proliferation and drug resistance in the breast cancer cells. (**A**–**B**) Influence of IGF2 with different concentrations on MCF‐7 and SKBR3 cell proliferation. Cell proliferation was analysed by MTT assay. (**C**–**D**) Cell proliferation of MCF‐7 and SKBR3 treated with IGF2 (50 ng/ml) and paclitaxel (16 nM) by colony formation assay. (**E**–**F**) Apoptosis was assayed in breast cancer cell with IGF2 or paclitaxel treatment by flow cytometry. (**G**) Changes of cell proliferation and apoptosis‐associated molecules in the breast cancer cells with IGF2 or paclitaxel treatment. The data presented are shown as means ± S.D. collected from three independent experiments. *P <* *0.05, **P <* *0.01.

IGF2, as a growth factor, may involve in cell proliferation. So, we used IGF2 to treat breast cancer cells to observe cell proliferation and drug resistance. Breast cancer cells were treated with IGF2 (0, 5, 10, 50 and 100 ng/ml) and cell proliferation was assayed by MTT method. Cell survival rate was increased in dose‐dependent manner (Fig. 4A and B). About 50 ng/ml IGF2 was used for paclitaxel response, and it was observed that IGF2 could significantly enhance cell survival ability of MCF‐7 and SKBR3 cells (Fig. 4C and D). When MCF‐7 and SKBR3 cells were treated with IGF2, the cell apoptosis rates decreased compared with the cells with paclitaxel treatment, which suggested that IGF2 could protect breast cancer cell from apoptosis induced by paclitaxel MCF‐7 and SKBR3 cells (Fig. 4E and F). To further know the molecular mechanism of the above phenomena, cell apoptosis‐associated protein was examined by Western blotting. IGF2 treatment induced the up‐regulation of Bcl‐2 and Bax and down‐regulation of caspase‐3. Paclitaxel could inhibit the up‐regulation of IGF2‐induced protein changes (Fig. 4G).

### CD44^+^Fbs modulates Hedgehog signal pathway in the breast cancer cells *via* IGF2

**Figure 5:**
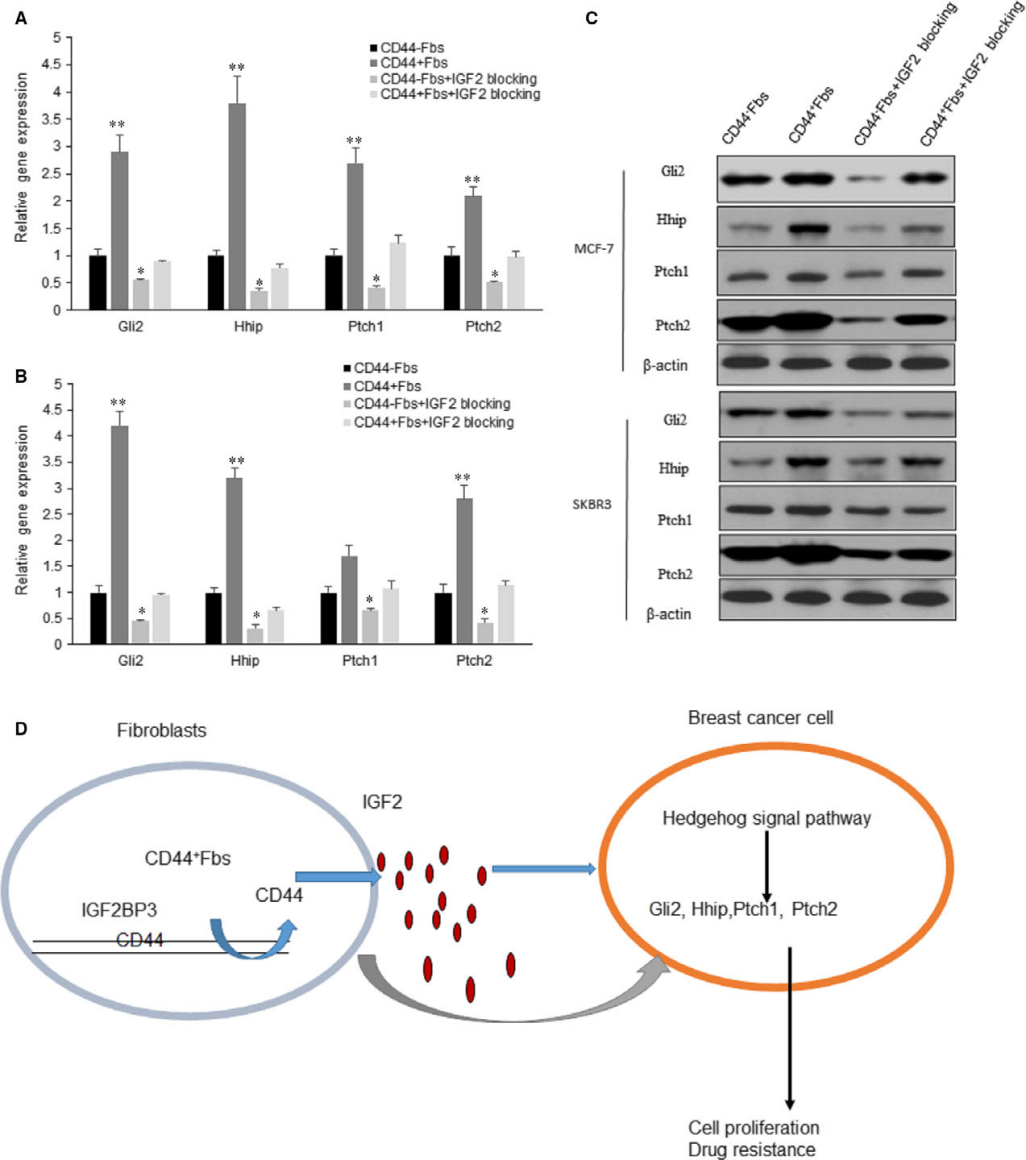
CD44^+^CAFs modulate Hedgehog signal pathway in the breast cancer cells *via* IGF2. (**A**–**B**) qRT‐PCR analysis of target gene expression of Gli2, Hhip, Ptch1 and Ptch2 of Hedgehog signal pathway in MCF‐7 and SKBR3 cells cocultured with CD44^+^CAFs or CD44^−^CAFs or IGF2 blocking. (**C**) Western blotting analysis of target gene expression of Gli2, Hhip, Ptch1 and Ptch2 of Hedgehog signal pathway in MCF‐7 and SKBR3 cells cocultured with CD44^+^CAFs or CD44^−^CAFs or IGF2 blocking. (**D**) CD44^+^CAFs could promote breast cancer cell proliferation and drug resistance by activating Hedgehog pathway *via* IGF2 secretion. The data presented are shown as means ± S.D. collected from three independent experiments. *P <* *0.05, **P <* *0.01.

To investigate the signal pathways that involves in CD44^+^Fbs induced breast cancer drug resistance, MCF‐7 and SKBR3 cells were cocultured with CD44^+^Fbs or CD44^−^Fbs. qRT‐PCR was used to target gene expression of Gli2, Hhip, Ptch1 and Ptch2 expression of Hedgehog pathway. Gli2, Hhip, Ptch1 and Ptch were expressed higher in the CD44^+^Fbs‐treated MCF‐7 and SKBR3 cells, while decreased in the cells with IGF2 blocked (Fig. 5A and B). Western blotting analysis showed that Gli2, Hhip, Ptch1 and Ptch protein levels of Hedgehog signal pathway were activated in the CD44^+^Fbs‐treated MCF‐7 cells (Fig. 5C). This indicated that CD44^+^Fbs could promote breast cancer cell proliferation and drug resistance by activating Hedgehog pathway *via* IGF2 secretion (Fig. 5D).

## Discussion

CD44 is usually used as one of the stem cell markers of some cancers including breast, lung, prostate, ovarian, cervical and colorectal cancers and neuroblastoma 15, 16. CD44 functions as a member of cell adhesion molecules that is responsible for mediating communication and adhesion between adjacent cells and the extracellular matrix (ECM) 17, 18. Except for cancer cells, fibroblasts express CD44.

Recent studies showed that CAFs express high CD44, which is important for maintaining cancer cell stemness 19. However, the roles of CD44 are lack of investigation in tumour stroma like fibroblasts. This report showed stromal CD44 plays a role in maintaining the stemness of CSCs by comparing CAFs from tumours induced in wild‐type or CD44‐mutant mice using CSC models. It is found that CD44‐positive CAFs are detected in both vascularized and non‐vascularized areas of tumours and seem to connect with CD31‐positive endothelial cells. Therefore, it remains possible that blood vessels provide their intraluminal nutrient to cancer cells in avascular area stemness 19. In this study, we cocultured CD44^+^Fbs or CD44^−^Fbs and breast cancer cells and found that CD44^+^Fbs could promote breast cancer cell proliferation and suppress cell apoptosis induced by paclitaxel. This suggests that CD44 on fibroblasts protects breast cancer cells from death.

The molecular mechanism of the above results demonstrated that IGF2BP3 bound to CD44 mRNA sequence and then promoted CD44 expression in fibroblasts. The result was the same to a previous study in myeloma cells 20. The binding of IGF2BP3 and CD44 is also related to IGF2 expression. Our data indicated that the conditioned medium of cocultured CD44^+^Fbs produced more IGF2 than CD44^−^Fbs. In order to confirm this, expression levels of IGF2 protein levels in breast cancer cells with or without CD44^+^Fbs or CD44^−^Fbs were examined by Western blotting and IGF2 indeed expressed much higher in breast cancer cells cocultured with CD44^+^Fbs than with CD44^−^Fbs. When breast cancer cells were exposed to IGF2, cell growth was accelerated and apoptosis induced by paclitaxel was prevented. So, it is very clear that IGF2 plays an important role in breast cancer proliferation and drug resistance.

Previous reports showed that IGF2 expression enhanced in basal cell carcinoma and breast cancer stroma 12, 13, which suggests IGF2 plays important roles in breast cancer microenvironment. IGF2 is an important growth factor which may involves in various signal pathways, but its signal pathway in cells is less known. In our study, we found that IGF2 could activate Hedgehop signal pathway in the breast cancer cells in the presence of CD44^+^Fbs. As per the data shown in the results, cyclin D1, Bcl‐2, Bax and caspase‐3 are the downstream genes of PI3K‐AKT. Importantly, we found that IGF2 secretion from CD44^+^Fbs activates Hedgehog signal pathway to increase breast cancer cell growth and induce drug resistance. IGF2 could modulate breast cancer cell biological behaviours in tumour stroma.

In conclusion, we demonstrated the role of CD44 expressed on fibroblasts in tumour microenvironment. CD44 was abundantly expressed on fibroblasts and CD44^+^Fbs and CD44^−^Fbs were isolated. Using Transwell coculture system, we found that breast cancer cell proliferation was enhanced in the presence of CD44^+^Fbs than CD44^−^Fbs. It was shown breast cancer cells cocultured with CD44^+^Fbs became more resistance to paclitaxel and lower apoptosis rates than cells cocultured with CD44^−^Fbs. The mechanism of the above phenomena may be that IGF2BP3 combines CD44 which promoting IGF2 secretion in fibroblasts and then activates Hedgehog signal pathway in breast cancer cells. The study suggests that CD44 and IGF2 are potential targets for breast cancer therapy.

## Conflict of interest

The authors confirm that there are no conflict of interests.

## References

[1] Friedl P , Alexander S . Cancer invasion and the microenvironment: plasticity and reciprocity. Cell. 2011; 147: 992–1009. 22118458 10.1016/j.cell.2011.11.016

[2] Laberge RM , Awad P , Campisi J , *et al* Epithelial‐mesenchymal transition induced by senescent fibroblasts. Cancer Microenviron. 2012; 5: 39–44. 21706180 10.1007/s12307-011-0069-4 PMC3343197

[3] Giaccia AJ , Schipani E . Role of carcinoma‐associated fibroblasts and hypoxia in tumor progression. Curr Top Microbiol Immunol. 2010; 345: 31–45. 20517716 10.1007/82_2010_73

[4] Duda DG , Ancukiewicz M , Isakoff SJ , Krop IE , Jain RK . Seeds and soil: unraveling the role of local tumor stroma in distant metastasis. J Natl Cancer Inst. 2014; 106(8): ePub ‐ PMID: 25082335. 10.1093/jnci/dju187 25082335

[5] Franco OE , Shaw AK , Strand DW , *et al* Cancer associated fibroblasts in cancer pathogenesis. Semin Cell Dev Biol. 2010; 21: 33–39. 19896548 10.1016/j.semcdb.2009.10.010 PMC2823834

[6] Cammarota F , Laukkanen MO . Mesenchymal Stem/Stromal Cells in Stromal Evolution and Cancer Progression. Stem Cells Int. 2016; 2016: 4824573. 26798356 10.1155/2016/4824573 PMC4699086

[7] Lee C , Jia Z , Rahmatpanah F , *et al* Role of the adjacent stroma cells in prostate cancer development and progression: synergy between TGF‐β and IGF signaling. Biomed Res Int. 2014; 2014: 502093. 25089270 10.1155/2014/502093 PMC4095744

[8] Karagiannis GS , Poutahidis T , Erdman SE , *et al* Cancer‐associated fibroblasts drive the progression of metastasis through both paracrine and mechanical pressure on cancer tissue. Mol Cancer Res. 2012; 10: 1403–1418. 23024188 10.1158/1541-7786.MCR-12-0307 PMC4399759

[9] Viola A , Sarukhan A , Bronte V , *et al* The pros and cons of chemokines in tumor immunology. Trends Immunol. 2012; 33: 496–504. 22726608 10.1016/j.it.2012.05.007

[10] Smith AL , Robin TP , Ford HL . Molecular pathways: targeting the TGF‐β pathway for cancer therapy. Clin Cancer Res. 2012; 18: 4514–4521. 22711703 10.1158/1078-0432.CCR-11-3224

[11] Otranto M , Sarrazy V , Bonté F , *et al* The role of the myofibroblast in tumor stroma remodeling. Cell Adh Migr. 2012; 6: 203–219. 22568985 10.4161/cam.20377 PMC3427235

[12] Szabó P , Kolář M , Dvořánková B , *et al* Mouse 3T3 fibroblasts under the influence of fibroblasts isolated from stroma of human basal cell carcinoma acquire properties of multipotent stem cells. Biol Cell. 2011; 103: 233–248. 21355851 10.1042/BC20100113

[13] Rasmussen AA , Cullen KJ . Paracrine/autocrine regulation of breast cancer by the insulin‐like growth factors. Breast Cancer Res Treat. 1998; 47: 219–233. 9516078 10.1023/a:1005903000777

[14] Hedborg F , Fischer‐Colbrie R , Ostlin N , *et al* Differentiation in neuroblastoma: diffusion‐limited hypoxia induces neuro‐endocrine secretory protein 55 and other markers of a chromaffin phenotype. PLoS ONE. 2010; 5. 10.1371/journal.pone.0012825 PMC2941466 20862257

[15] Inoue K , Fry EA . Aberrant Splicing of Estrogen Receptor, HER2, and CD44 Genes in Breast Cancer. Genet Epigenet. 2015; 7: 19–32. 26692764 10.4137/GEG.S35500 PMC4669075

[16] Schwertfeger KL , Cowman MK , Telmer PG , *et al* Hyaluronan, Inflammation, and Breast Cancer Progression. Front Immunol. 2015; 6: 236. 26106384 10.3389/fimmu.2015.00236 PMC4459097

[17] Louderbough JM , Schroeder JA . Understanding the dual nature of CD44 in breast cancer progression. Mol Cancer Res. 2011; 9: 1573–1586. 21970856 10.1158/1541-7786.MCR-11-0156

[18] Götte M , Yip GW . Heparanase, hyaluronan, and CD44 in cancers: a breast carcinoma perspective. Cancer Res. 2006; 66: 10233–10237. 17079438 10.1158/0008-5472.CAN-06-1464

[19] Kinugasa Y , Matsui T , Takakura N . CD44 expressed on cancer‐associated fibroblasts is a functional molecule supporting the stemness and drug resistance of malignant cancer cells in the tumor microenvironment. Stem Cells. 2014; 32: 145–156. 24395741 10.1002/stem.1556

[20] Canella A , Cordero Nieves H , Sborov DW , *et al* HDAC inhibitor AR‐42 decreases CD44 expression and sensitizes myeloma cells to lenalidomide. Oncotarget. 2015; 6: 31134–31150. 26429859 10.18632/oncotarget.5290 PMC4741593

